# Milk and meat consumption patterns and the potential risk of zoonotic disease transmission among urban and peri-urban dairy farmers in Ethiopia

**DOI:** 10.1186/s12889-022-12665-4

**Published:** 2022-02-03

**Authors:** Tilaye Teklewold Deneke, Adam Bekele, Henrietta L. Moore, Tadele Mamo, Gizat Almaw, Getnet Abie Mekonnen, Adane Mihret, Rea Tschopp, Likawent Yeheyis, Catherine Hodge, James L. N. Wood, Stefan Berg, the ETHICOBOTS consortium, Abraham Aseffa, Adane Mihret, Bamlak Tessema, Bizuneh Belachew, Eshcolewyene Fekadu, Fantanesh Melese, Gizachew Gemechu, Hawult Taye, Rea Tschopp, Shewit Haile, Sosina Ayalew, Tsegaye Hailu, Rea Tschopp, Adam Bekele, Chilot Yirga, Mulualem Ambaw, Tadele Mamo, Tesfaye Solomon, Tilaye Teklewold, Solomon Gebre, Getachew Gari, Mesfin Sahle, Abde Aliy, Abebe Olani, Asegedech Sirak, Gizat Almaw, Getnet Mekonnen, Mekdes Tamiru, Sintayehu Guta, James Wood, Andrew Conlan, Alan Clarke, Henrietta L. Moore, Catherine Hodge, Constance Smith, R. Glyn Hewinson, Stefan Berg, Martin Vordermeier, Javier Nunez-Garcia, Gobena Ameni, Berecha Bayissa, Aboma Zewude, Adane Worku, Lemma Terfassa, Mahlet Chanyalew, Temesgen Mohammed, Miserach Zeleke

**Affiliations:** 1 Amhara Regional Agricultural Research Institute, Bahar Dar, Ethiopia; 2 Ethiopian Institute of Agricultural Research, Addis Ababa, Ethiopia; 3 Institute for Global Prosperity, University College London, London, UK; 4 National Animal Health Diagnostic and Investigation Center, Sebeta, Ethiopia; 5 Armauer Hansen Research Institute, Addis Ababa, Ethiopia; 6 Swiss Tropical and Public Health Institute, Basel, Switzerland; 7 University of Basel, Basel, Switzerland; 8 Cambridge University, Cambridge, UK; 9 Animal and Plant Health Agency, Weybridge, UK

**Keywords:** Zoonosis, Raw meat, Raw milk, Consumption, Bovine tuberculosis, *Mycobacterium bovis*

## Abstract

**Background:**

In the Ethiopian dairy farming system, prevalence of zoonotic diseases such as bovine tuberculosis (bTB) is high in the cattle population. This, combined with some risky milk and meat consumption habits, such as raw milk and uninspected raw meat consumption, poses a considerable risk of zoonotic disease transmission. A survey was conducted to investigate milk and meat consumption patterns, and the level of exposure to urban and peri-urban dairy-keeping households for risks of zoonotic disease transmission.

**Methods:**

Data on milk and meat consumption behaviours and other socioeconomic and demographic variables were collected from 480 urban and peri-urban dairy farms randomly surveyed in major towns in Ethiopia (Mekele, Hawassa, and Gondar towns, Addis Ababa city, as well as five Oromia towns around Addis Ababa). Determinants of raw milk consumption associated with a number of demographic and socio-economic factors were analysed using a generalised ordered logistic model.

**Results:**

The results indicated that about 20% the population consumed raw milk and their awareness about pasteurisation and its benefits were low. Location, gender of the household head, previous bTB testing of cattle on the farm, knowledge of zoonotic risks associated with raw milk consumption, household size, and per-capita milk consumption were found to be important determinants of the frequency of raw milk consumption. About 60% of the respondents were exposed to the risk of zoonotic diseases through their habit of frequently consuming raw meat. This was despite that over 90% of the respondents were aware of possible zoonotic risks of raw meat consumption. The determinants of raw meat consumption behaviours were associated with location, gender and age of the household head, household size, meat type preference, per-capita meat consumption, knowledge about disease transmission risks, and training on zoonoses.

**Conclusion:**

Creating awareness about the risk factors for zoonotic transmission of diseases through training and media campaigns, improving meat hygiene through better abattoir services, and inducing behavioural change around meat sourcing, raw meat and raw milk consumption, are all crucial to the successful prevention and control of the spread of zoonotic diseases, including bTB.

**Supplementary Information:**

The online version contains supplementary material available at 10.1186/s12889-022-12665-4.

## Background

Ethiopia is an agrarian country with Africa’s largest national livestock herd and over 70% of the human population are directly engaged in the agricultural sector. In such settings, peoples have close interaction with animals and animal products. Considering the high burden of zoonotic diseases in the Ethiopian livestock [1], the community is at risk of zoonotic transmission through inhalation and ingestion of pathogens. Consumption of uncooked or unprocessed food increases the risk for disease transmission of bovine tuberculosis (bTB), bovine leukosis, brucellosis, anthrax, campylobacteriosis, meningitis, typhoid fever, and gastroenteritis and the like [2–5].

The aim of this paper was to investigate milk and meat consumption patterns among urban and peri-urban dairy-keeping households in Ethiopia, in order to understand the level of exposure of these segments of society to the transmission risk of bTB and other zoonotic diseases through their milk and meat consumption habits. Studying these issues and the socioeconomic factors that determine the milk and meat consumption behaviour of dairy farmers, and consumers more generally, are important for devising strategies for controlling the spread of zoonotic diseases such as bTB, and therefore it was taken on by the Ethiopia Control of Bovine Tuberculosis Strategies (ETHICOBOTS) project - a collaboration between researchers in the United Kingdom, Switzerland and Ethiopia - with the purpose to improve the scientific knowledge base on bTB and to explore control strategies for the disease in Ethiopia.

Although many high income countries are free of bTB due to comprehensive test-and-slaughter programmes [6], the disease is endemic in cattle in many other parts of the world [7]. The latter is also the case for Ethiopia in which vast epidemiological studies have shown its presence in most parts of the country [8] and the burden is particularly high among intensively reared dairy cattle kept in urban and peri-urban settings [9–11]. National test-and-slaughter programmes with the purpose to eradicate animal and zoonotic diseases are costly and often not prioritised in low income countries such as Ethiopia.

The main causative agent of bTB is the bacterium *Mycobacterium bovis* (*M. bovis*), causing TB disease in a wide range of animals as well as in humans. However, as the name ‘bovine TB’ infers, cattle are considered as the main reservoir of this disease. The most common routes for zoonotic transmission of bTB are by ingestion, aerosol inhalation, or direct contact with mucous membranes and skin abrasions [6]. Airborne transmission is often associated with respiratory disease manifestation while ingestion of infected food (i.e. milk- or meat-borne) is linked to increase risk of extra-pulmonary tuberculosis [12].

*Raw milk consumption*. Milk offers a huge potential to improve nutrition and livelihoods for hundreds of millions of poor people and it is estimated that some 750 million people are engaged in milk production around the world, the majority of whom are in low and middle income countries [13]. In countries that are transforming from an agrarian and rural society towards an increased urban and ‘modern’ society, milk production often shifts from small scale cattle farming towards larger intensive dairies. The latter are associated with an increased risk for transmission of infectious diseases, including bTB [8, 9, 11, 14]. In addition, pasteurisation plants in low income countries are limited and the habit of raw milk consumption is widespread. Thereby, these factors create the perfect conditions for increased risks of zoonotic transmission of bTB through raw milk consumption, especially among dairy farmers with bTB positive animals.

In many societies around the world, raw (unpasteurised or unboiled) milk consumption is a deeply rooted cultural habit. In the Ethiopian context, milk is often consumed in its natural state or as a fermented form [15]. Some even believe that boiling or pasteurising processes destroy the quality of the milk [16, 17].

Studies have shown that *M. bovis* has frequently been isolated from unpasteurised and un-boiled milk samples [18–20]. In countries where pasteurisation is not widely practiced and where there is poor milk hygiene and a common habit of raw milk consumption, it has for long been estimated that about 10–15% of all human TB cases are caused by *M. bovis* [21]. Although such high rates of zoonotic transmission may exist in certain contexts and under certain conditions, more recent reports suggest that the global impact on human TB by *M. bovis* is around 1.5% [7], much lower than previously estimated.

*Raw meat consumption*. Meat consumption in Ethiopia is also a deep-rooted cultural behaviour. Meat is often consumed as part of the staple diet of the people and also during special occasions of festivity. Its cultural symbolic weight is greater than any other food [22]. Eating raw meat or half cooked meat is very common and although Ethiopians from various cultures enjoy eating meat, they are generally very selective, in that only poultry, beef, mutton, goat and fish (not including shell-fish) are culturally and religiously acceptable. Eating other kinds of meat, such as pork, is a cultural taboo among most Ethiopians [22] while camel meat is allowed in Muslim communities [23].

In high income countries, where bTB control and/or eradication programmes and effective meat inspection mechanisms usually are in place, the risk of getting infected with bTB due to meat consumption is very low [6]. This is because viable *M. bovis* have rarely been isolated from skeletal muscles and the risk of getting TB from meat is apparently not from infectivity of muscle meat but rather from possible contamination of meat surfaces during unhygienic slaughtering of animals with severe TB lesions in other organs [6, 24]. An effective meat inspection would condemn any such carcass and protect the consumers from risk of infection. In addition, the *M. bovis* bacterium is sensitive to heat and any contamination could be deactivated by cooking [25]. In low income countries though, where meat inspection mechanisms are likely to be less effective and where the vast majority of the meat consumed is sourced from informal sources without disease inspection and proper hygiene, the habit of raw meat consumption could be a considerable risk factor for zoonotic transfer of bTB and other diseases [26]. It also needs to be said that since diseases such as bTB and brucellosis are often not controlled at farm level in low income countries, it is more likely that diseased cattle will reach the point of slaughter without such diseases having been detected or communicated.

In Ethiopia, there are three sources of meat for home consumption: purchase from local butchery (sourced from abattoir), home slaughter, or communal slaughter. There are no official estimates of the proportions of these meat sources, but as the abattoir service is very limited in Ethiopia (even the capital, Addis Ababa, has only one municipal abattoir service facility), people rely mostly on home or communal slaughter [27]. Communal slaughter is even a cultural tradition where approximately 4–10 neighbours or close friends get together to buy an animal and slaughter it for holidays or other festive events.

Butcheries as a source of meat, which usually obtain its meat from the official abattoirs, is likely the safest way of sourcing meat. However, it carries also the risk of zoonotic disease transmission as meat inspection in abattoirs in Ethiopia is limited. A study on routine abattoir inspection in Ethiopia detected only 55% of all cattle confirmed with TB lesions [28], suggesting that meat infected with *M. bovis* have a chance to enter into the food chain. Biffa et al. [29] also indicated that inspection techniques used at Ethiopian abattoirs failed to detect 70% of the carcasses with grossly-visible lesions of TB.

Unlike in official abattoirs in Ethiopia, backyard slaughter does not undergo any formal meat inspection. Therefore, sourcing meat through home and communal slaughter could be potential risk factors for zoonotic disease transmission. In fact, focus group discussions with Ethiopian farmers have revealed that slaughter is a common mechanism for dealing with chronically sick animals [30]. Home slaughter is more readily practiced on small ruminants. Slaughter of large livestock species is often due communally as a means of providing financial assistance to the owner, as each participant will pay money to the animal owner in return for a share of the meat. This sharing praxis makes communal slaughter potentially the most risky meat source in terms of zoonotic transfer of diseases between households and provide more opportunities for disease transmission through consumption of infected meat. In addition to sharing the meat between households, feasting together on raw meat and organs such as liver and kidneys are widespread practices in Ethiopia [22], and conducted as part of the communal slaughtering tradition.

With this background we set out to collected data on different aspects of milk and meat consumption e.g. sourcing preferences, type preferences, and consumption behaviours as well as the determinants thereof. We focused on dairy farmers in urban and peri-urban dairy production areas where the prevalence of certain zoonotic diseases such as bTB have been reported to be particular high [10, 11, 31–34], to better understand their consumption behaviour in relation to risks for zoonotic disease transmission.

## Methods

### The study areas

**Fig. 1:**
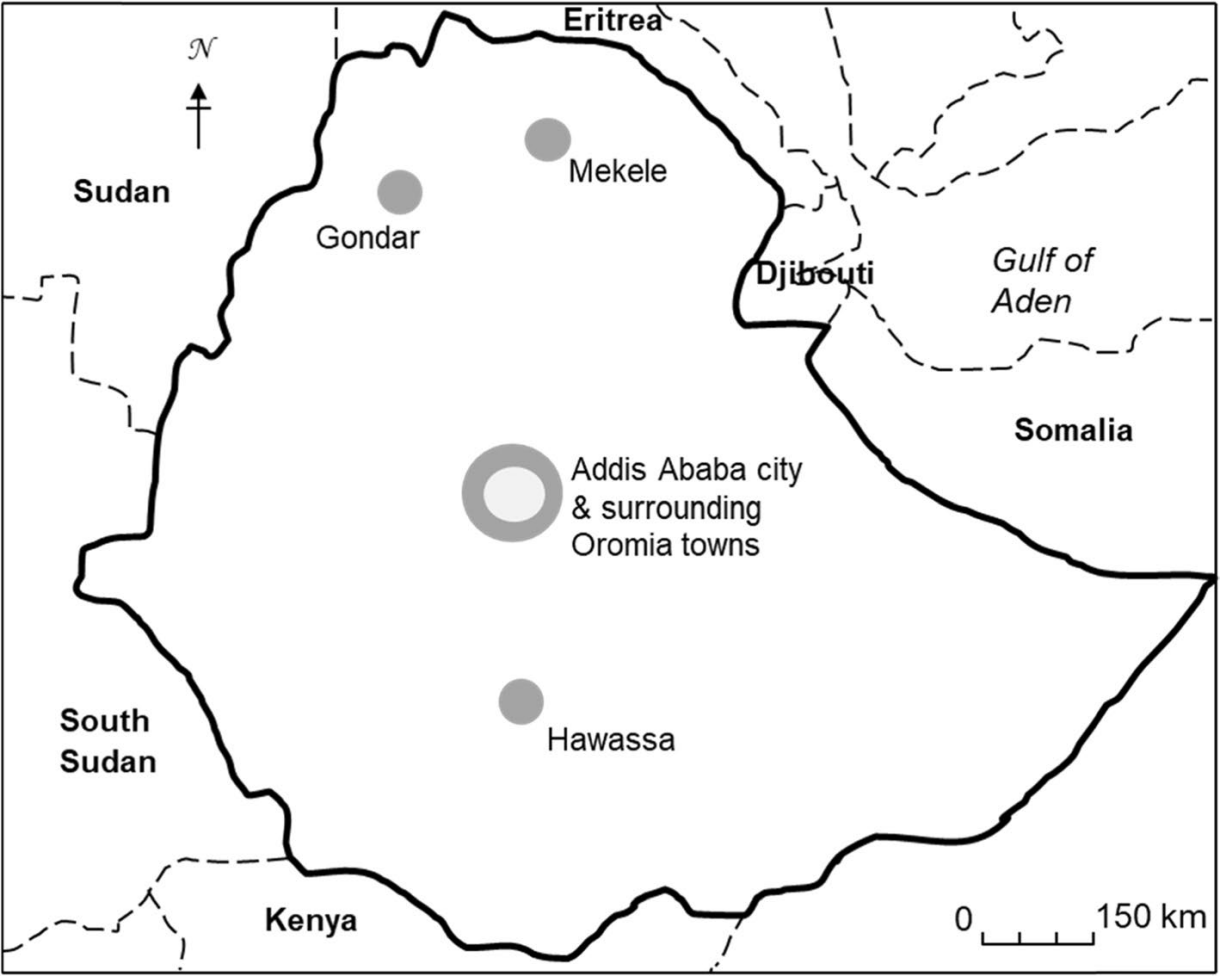
Geographical locations of the study sites in Ethiopia included in this survey. Study sites of Addis Ababa city and surrounding Oromia towns are shown as two overlapping dots on the map. Dot-size do not represent actual sample-size as shown in Table 1

The study was carried out in five study sites (Fig. 1) that included Mekele, Hawassa, and Gondar towns, Addis Ababa city, as well as five Oromia towns (Holeta, Sebeta, Debre Zeit, Sululta and Sendafa) surrounding the capital and defined as one single study site (Addis Ababa surroundings). The study areas contained both urban and peri-urban settings where intensive dairy-farming activities took place. In these intensive dairy systems, Holstein-Frisian (H-F) cattle and their crosses with local zebu breeds were mainly kept with indoor feeding and zero grazing. The number of animals in the recruited farms ranged from five to over 100 dairy cattle and all were kept for commercial milk production purpose. All the study sites are mid-altitude areas ranging from 1500 to 2500 m above sea level with mild temperatures and annual rain fall between 800 and 1500 mm. Addis Ababa is a big city with a population around 3.5 million and its surrounding suburban areas, where a sprawling intensive dairy farming is practiced, have also a large population estimated to around 1.5 million people [35]. Mekele, Hawassa, and Gondar are regional towns each having a population ranging between 300,000–700,000 people. The total dairy animal population in Ethiopia with exotic blood (mainly Pure exotic blood and exotic blood Zebu crosses) was estimated to be around 1.44 million [36], of which the majority is found in these study sites. According to the last few years’ trend, this figure is likely to continue increasing in the coming years due to an artificial insemination programme by the Government and the increase in demand for milk and meat in the country [37].

### The sample farms

We interviewed 480 urban and peri-urban dairy farmers about their milk and meat consumption habits as part of a wider cross-sectional study of the epidemiology of bTB in Ethiopia, described in Almaw et al. and Mekonnen et al. [9, 11]. In short, households/farms were selected using a two-stage stratified sampling technique, where in the first stage five urban/peri-urban study sites were selected (see ‘The Study Areas’ above and Fig. 1). In the second stage, using random numbers, a random sample of farmers was selected from a sampling frame composed of list of farms in the areas obtained from the local agriculture office. Farm size and estimated total number of farms in each study site were taken in to consideration to make the sample proportional to size. Table 1 shows the distribution of sample farms at each study site.

**Table 1:** Distribution of sample farms by site and herd size

Site	Herd size of dairy cattle			Total
	small-holder farm 5–19	medium-size farm 20–49	large-size farm > 49	
Addis Ababa city	123	34	7	164
Oromia towns around Addis Ababa	82	36	20	138
Gondar	53	9	4	66
Mekele	50	8	2	60
Hawassa	29	19	4	52
Total	337	106	37	480

### Data collection

Survey data was collected through a well designed and tested questionnaire by trained, both male and female, enumerators with the supervision of ETHICOBOTS researchers. Along with survey questions, the cattle in the 480 sampled dairy herds were tested for bTB using the Single Intradermal Comparative Cervical Tuberculin (SICCT) test [38] with PPD-A and PPD-B sourced from Lelystad (The Netherlands); the results of these tests have been published elsewhere [9, 11] but utilised for analyses in the current study. The questionnaire was prepared and tested by a multidisciplinary team composed of agricultural economists, anthropologists, veterinarians and other biomedical scientists. Computer Assisted Personal Interview (CAPI) equipment was used for data collection. The questionnaire was designed to collect socioeconomic characteristics of farmers and farm workers, farm management practices, knowledge and attitude towards bTB and other zoonotic diseases as well as milk and meat consumption behaviour of farmers, farm workers and their families. Farm owners or managers were the respondents during the interview which typical took about one and half hours. In only one case a farmer refused to answer all the questions and interrupted and turned back on the enumerator; otherwise the farmers were cooperative and willing to answer the questions as well as allowing bTB testing of their animals.

### Data analysis: generalised ordered logistic model

Descriptive and inferential statistical analysis tools were used to analyse the data collected. In order to analyse the determinants of milk and raw meat consumption frequency we used generalised ordered logit model described below.

The ordered logit is a widely used model when dealing with outcome variable measured at ordinal scale. The assumption that ordered logit makes is that the gap among the various scales of the ordering are equidistant to each other. It is assumed that the effect of each predictor across the categories of the ordinal dependent variable is the same and is known as proportional odds assumption [39–41]. However, this does not often hold up [39, 42]. In our data, Brants’ Wald test [43] reveals that the effect of each explanatory variable varies across different cutting points of the ordinal outcome variable; as a result, we adopted generalised ordered logit model that relaxes proportional odds assumption [42, 44]. The generalised ordered logit can estimate models (i.e. partial proportional odds) that are more parsimonious than non-ordinal alternatives, such as multinomial logit (however, multinomial logit ignores the ordering of categories and hence would not be more appropriate to deal with ordered outcomes). The model is estimated by using a constraint partial proportional odds model that entertain the violation of parallel assumption to all predictors and/or a certain predictors of the model [45, 46]. In such a way, there would be as much number of binary logit equations as the number of ordinal levels but one, where first it is category 1 versus all others, then categories 1 & 2 versus all others, then 1, 2 & 3 versus all others, etc. [42]. This is based on the assumption that the effect of the predictor variable may vary across the range of the predicted variable.

As discussed further above, raw milk consumption is often considered to be a risky behaviour in terms of zoonotic transmission of bTB and other diseases [2, 21, 33]. We hypothesized that raw milk consumption habit and its frequency are determined by demographic and socioeconomic variables such as study site, gender, age, literacy, income or wealth status, per capita consumption levels, as well as knowledge about the risk of zoonosis transfer of diseases. The dependent variable, ‘raw milk consumption frequency’, was measured as an ordinal variable with three ordinal scales, namely:

(A) No raw milk consumption;
(B) Moderate level of raw milk consumption (only occasional consumption of raw milk); and
(C) High frequency of raw milk consumption (at least once a fortnight)

We used Generalised Ordered Logit as that model was found to violate the proportional odds ratio assumption discussed in the methods section. As indicated in Table 4, *Eq. 1* compares the category of those farmers who never consumed raw milk (A) to the category of those who did (B + C). *Eq. 2* models the category of those who never consumed raw milk or who had an occasional consumption frequency (A + B) to those who had a high frequency consumption (C).

Similarly in order to analyse the determinants of raw meat consumption habits, we hypothesized that the habits and frequency of raw meat consumption among dairy farmers in Ethiopia could be determined by demographic and socioeconomic variables such as study site, gender, age, literacy, income or wealth status, as well as having knowledge about the risk of zoonotic disease transfer through raw meat consumption. To explore this hypothesis within our data set, the dependent variable ‘raw meat consumption frequency’ was measured as an ordinal variable with three ordinal scales, namely:

(IV) No raw meat consumption;
(V) Moderate level of raw meat consumption (less than once a fortnight); and
(VI) High frequency of raw meat consumption (at least once a fortnight)

We used generalised ordered logit as that model was found to violate the proportional odds ratio assumption. As shown in Table 4, *Eq. 3* compares the category of farmers who never consumed raw meat (D) to the category of those who did (E + F), while *Eq. 4* models the category who never consumed raw meat or had a low consumption frequency (D + E) to the category of high frequency consumers (F).

## Results

Below follows the results of our interviews on milk and meat consumption habits held with 480 urban and peri-urban dairy farmers from the five study sites shown in Fig. 1.

### Milk consumption

#### Per capita milk consumption

The average per capita milk consumption per day for our sample was found to be 0.25 l (SD = 0.26). Farmers were asked about their consumption on a daily and monthly rather than on a yearly basis, as the latter would be difficult to recall and estimate and could also be erroneous due to there being many fasting days in the Ethiopian orthodox church calendar when believers do not consume milk. In this sample the orthodox Christians made up 83.3%. This average per capita milk consumption figure is statistically significantly higher (t = 16.09; *P* < 0.001) than the national average of 19 l per year, which corresponds to ~ 0.05 l per day [47]. The mean per capita milk consumption among the sample was not found to be statistically significant between sexes, religions, literacy statuses, or study sites (Supplementary Table S1).

#### Raw milk consumption

Farmers were asked about their habits of raw (unboiled and unpasteurised) milk consumption. As shown in Table 2A, 77.5% of the respondents (*n* = 371) indicated that they never drank raw milk while about 20.4% drank raw milk but with varying degrees of frequency. Only 8.1% (*n* = 39) stated that they were regular drinkers of raw milk, drinking it at least once a day. Although the majority of the sample farmers indicated that they did not drink raw milk, about 82% of the respondents did actually drink fermented milk, a yoghurt commonly called *ergo* in the Amharic language, which is usually made from non-pasteurised/unboiled milk.

**Table 2:** Milk consumption characteristics among sampled dairy farmers in Ethiopia

A. Consumption frequency of raw and processed milk reported by dairy farmers						
	Raw milk		Pasteurised milk		Boiled milk	
	n (%)	Cum. %	n (%)	Cum. %	n (%)	Cum. %
Everyday	39 (8.1)	8.1	3 (0.6)	0.6	122 (25.5)	25.5
3–6 times a week	30 (6.3)	14.4	9 (1.9)	2.5	222 (46.3)	71.8
Once/twice a week	14 (2.9)	17.3	6 (1.3)	3.8	83 (17.3)	89.1
Once/twice a month	15 (3.1)	20.4	9 (1.9)	5.7	18 (3.8)	92.9
On Special occasions only	10 (2.1)	22.5	26 (5.4)	11.1	11 (2.3)	95.2
Not at all	371 (77.5)	100	426 (88.9)	100	23 (4.8)	100
Total	479 (100)		479 (100)		479 (100)	
B. Farmers’ perception of the healthiness of drinking raw milk						
How healthy is drinking raw milk?	n (%)		Cum. %			
Very healthy	23 (4.8)		4.8			
Healthy	48 (10.1)		14.9			
Do not know	35 (7.4)		22.3			
Unhealthy	238 (50.1)		72.4			
Very unhealthy	131 (27.6)		100			
Total	475 (100)					

We also investigated raw milk consumption in relationship to gender, literacy, study site, religion, and age and we found that it was not related to any of these socioeconomic variables, except for study site (data not shown). A statistically significant systematic relationship between study site and raw milk consumption habit was established (likelihood-ratio chi2 (4) = 28.70, *P* < 0.001) with only 5% of the dairy farmers from Mekele (in Northern Ethiopia) indicated that they consumed raw milk as compared to 37% of those from Hawassa (in the South).

The majority of respondents (88%; *n* = 424) indicated that they knew that drinking raw milk can cause diseases while only 5.6% (*n* = 27) indicated that it does not cause any diseases. Some 6.0% (*n* = 29) indicated that they did not know about such risk (data not shown), while 78% indicated that they thought drinking raw milk to be unhealthy or very unhealthy (Table 2B).

Neither general training on zoonosis transmission mechanisms (Fisher’s exact = 0.415), nor specific training on bTB bore any relation to raw milk consumption frequency (Fisher’s exact = 0.680). Moreover, we observed no difference in the frequency of raw milk consumption between those farms whose animals tested positive for bTB before our survey and those which were not. This indicates that acquiring knowledge of the bTB status of the cattle at farm level has not generally led to change in raw milk consumption behaviour of these farmers.

Despite their knowledge of the possible risk of disease transmission, a considerable number of our sample (20.4%; *n* = 98) consumed raw milk frequently and on a regular basis (Table 2A). Among participants drinking raw milk, 47% had bTB positive animals in their herd but there was in fact no statistically significant difference in the raw milk consumption habits between farms with bTB positive and negative cattle. Interestingly however, we did find that there was a statistically significant relationship between raw milk consumption habit and occurrence of TB disease in the farm household in the last 3 years before the survey (likelihood-ratio chi2(1) = 12.09; *P* = 0.001). Among those farm households which reported a TB case in the last 3 years, 41% indicated that they were in the habit of consuming raw milk, compared to only 20% among those farmers who reported no TB cases. This result warrants further clinical epidemiological investigation to establish whether the confirmed TB cases might be attributable to zoonotic or human TB, caused by *M. bovis* or *M. tuberculosis*, respectively.

#### Pasteurised milk consumption

As shown in Table 2A, nearly 89% of the surveyed farmers did not drink pasteurised milk. Only 38% (*n* = 181) knew the benefits of pasteurisation, while 54% (*n* = 259) did not and 8.1% (*n* = 39) had never heard about pasteurisation. Only 1.5% of the respondents indicated that their main source of milk was pasteurised milk. Pasteurised milk was ranked second by 7.6% respondents, 15% ranked it third and 19% as fifth (data not shown).

Investigation of the relationship between literacy and pasteurised milk consumption frequency showed no systematic relationship (Fisher’s exact test *P* value = 0.690). Since the majority of the large dairy farms and pasteurisation plants in Ethiopia are located in the capital Addis Ababa and its surrounding towns, it was logical to expect regional differences in the use of pasteurised milk consumption frequency. However, contrary to what was expected, no relationship was found between study sites and pasteurised milk consumption (Fisher’s exact test *P* value = 0.480). Similarly, there was not a statistically significant relationship between gender and frequency of pasteurised milk consumption (Fisher’s exact test *P* value = 0.156). It needs to be added though that the generally low levels of consumption of pasteurised milk among the surveyed farmers could be because they have easier access to unpasteurised milk than the average consumer. On top of this, our data show that there is statistically significant relation between bTB status of the farmers’ herd and knowledge about pasteurisation/pasteurised milk (Chi(2) = 7.19 and *P* = 0.007) i.e. among those farmers who had bTB positive cattle, 55% had no knowledge of pasteurisation and among those farmers who did not know about pasteurisation, 42% had bTB positive animals. These results are alarming given the high prevalence of zoonotic diseases in the area, including bTB.

#### Boiled milk consumption

Although the vast majority of respondents said they did not drink raw milk (78%) or pasteurised milk (89%), 89% (*n* = 427) of the respondents drank boiled milk at least once a week (Table 2A), while only 4.8% (*n* = 23) indicated that they never drank boiled milk. The frequency of boiled milk consumption was found to be dependent on study site (likelihood-ratio chi2(8) = 21.62; *P* = 0.006), with those in Hawassa (87%) and in Addis Ababa (75%) were drinking boiled milk more frequently than those in Gondar, in Amhara region (71%), in Mekele, in Tigray region (68%) and in the Oromia towns surrounding Addis Ababa (65%).

### Meat consumption

#### Per capita meat consumption

The published national average of per capita meat consumption in Ethiopia is 5.3 kg per annum [48], which corresponds to less than 0.5 kg per month. However, the corresponding consumption for urban areas is 11.5 kg (~ 1 kg per month). As shown in Table 3A, the latter figure is much in line with the mean rate of per capita monthly meat consumption of 1.25 kg (SD =1.44) among the dairy farmers in our sample, with Hawassa having the lowest (0.88 kg) and Addis Ababa having the highest (1.37 kg) consumption. Interestingly, the mean per capita meat consumption per month for male-headed households was found to be 1.35 kg, which was statistically higher (t = − 2.43, *P* = 0.015) than the 0.95 kg per month for female-headed households. We found no statistically significant difference in per capita meat consumption between study sites, religions (Christians and Muslims), or between households with illiterate and literate heads.

**Table 3:** Meat consumption characteristics among sampled dairy farmers in Ethiopia

A. Average monthly per capita meat consumption (in kg) among dairy farmers in each study site				
		Mean	SD	n (%)
Addis Ababa city		1.37	1.87	164 (36.7)
Oromia towns around Addis Ababa		1.12	1.06	135 (30.2)
Gondar		1.35	1.46	66 (14.8)
Mekele		1.33	0.95	57 (12.7)
Hawassa		0.88	0.49	25 (5.6)
Total		1.25	1.44	447 (100)
B. Frequency of meat consumption among sampled dairy farmers				
	**General meat consumption**		**Raw meat consumption**	
	n (%)	Cum. %	n (%)	Cum. %
Everyday	5 (1.0)	1.0	2 (0.4)	0.4
2–5 days a week	56.6 (271)	57.6	96 (20.0)	20.4
Once every fortnight	109 (22.8)	80.4	51 (10.7)	31.1
Once a month	66 (13.8)	94.2	66 (13.8)	44.9
Only for holidays	25 (5.2)	99.4	91 (19.0)	63.9
Never	3 (0.6)	100.0	173 (36.1)	100.0
Total	479 (100)		479 (100)	
C. Ranking order of meat source by sampled dairy farmers				
	**Rank 1**		**Rank 2**^**a**^	**Rank 3**^**a**^
	n (%)		n (%)	n (%)
Butchery	256 (53.9)		109 (24.0)	58 (17.8)
Home slaughter	174 (36.6)		191 (42.2)	91 (27.8)
Communal slaughter	45 (9.5)		153 (33.8)	178 (54.4)
Total	475 (100)		453 (100)	327 (100)
D. Farmers’ views about risks of getting disease while eating raw meat				
Do you think eating raw meat can cause diseases?			**Have you ever experienced diseases due to eating raw meat?**	
	n (%)		n (%)	
No	34 (7.1)		287 (59.9)	
Yes	445 (92.9)		192 (40.1)	
Total	479 (100)		479 (100)	

^a^ Not all respondents gave a second or third ranking

#### Meat type preference

For the whole sample, when respondents were asked to rank their meat type preference, it was found that 48% (*N* = 220) ranked beef as first choice, followed by mutton 32% (*n* = 144), chicken 11% (*n* = 48), and goat meat 9.4% (*n* = 43). However, these figures varied by region with a statistically significant association between preferred meat-type and study site (likelihood-ratio chi2(12) value of 135.5 and *P* < 0.001). The responding dairy farmers in Addis Ababa (62%) and in the towns of Oromia (64%), as well as in Hawassa (44%) tended to significantly prefer beef, while the majority in Gondar (75%) and Mekele (56%) in the Northern regions preferred mutton over any other meat. A similar association was observed between frequency of meat consumption and meat type preference, with Fisher’s Exact test of 0.029 being significant at the 0.05 level; i.e. those households which preferred beef tended to be more frequent meat consumers (79%). A one-way analysis of variance in terms of mean age of the respondents and their preferred meat type showed a statistically significant difference (F value = 5.49; *P* = 0.001). Those households which preferred chicken meat had the lowest mean age of 41 years (SD = 11) and this was significantly different from the mean age of 50 years (SD = 15) among those who preferred mutton and the mean age of 46 years (SD = 15) among those who preferred beef. Education, which was measured as a dummy variable with the two categories being literate or illiterate, showed no relationship to meat type preference (Fisher’s exact test *P* value = 0.830). We also tested for a relationship between gender of the household head and meat type preference but found no significant correlation.

#### Meat consumption frequency

As shown in Table 3B, only 1% of the responding farmers ate meat every day while the majority of them (57%) consumed meat 2–5 days a week. Only 0.6% (3 individuals) indicated that they did not consume meat at all. There was no statistical difference between study site and meat consumption frequency, and gender of the household head and meat consumption frequency. Also, no significant difference in mean age of respondents was observed between those households which frequently consumed meat and those who did so less frequently (t = − 0.2779 and *P* = 0.7812). On the other hand, literacy level and frequency of meat consumption were found to be associated (Fisher’s exact value *P* = 0.001); 56% of the illiterate household heads indicated a high frequency of meat consumption while about 83% of the literate households consumed meat at high frequency. 63.9% of the respondents habitually consumed raw meat (mainly beef) and about 20% were in the habit of consuming raw meat either every day or 2–5 times a week. However, more than a third of the respondents (36.1%) indicated that they had never consumed raw meat (Table 3B).

An investigation was conducted into the relationship between raw meat consumption frequency and demographic factors of which statistically significant associations were found between frequency and study site (likelihood-ratio chi2(20) = 120.6; *P* < 0.001) and religion (likelihood-ratio chi2(1) = 13.34; *P* < 0.001), respectively. Muslims tended to avoid raw meat, with 75% (15 out of 20) of the surveyed Muslims indicating that they had never consumed raw meat. Among the study sites surveyed, the proportion of dairy farmers who consumed raw meat more frequently (at least once in a fortnight) was 66% in Addis Ababa, 77% in Oromia, 66% in Hawassa, and 67% in Gondar. However, only 25% in Mekele had a habit of frequent raw meat consumption. No relationships between raw meat consumption frequency and gender, literacy, or age were found in this survey.

#### Meat source preference

As shown in Table 3C, the most preferred source of meat across the sample of farmers was found to be butchery (53.9%), followed by home slaughter (36.6%) and then communal slaughter (9.5%).

The relationship between meat source ranking and variables such as gender of the household head, literacy status, religion, study site, and age were examined. We found a statistically significant association between study site and the main source of meat (likelihood-ratio chi2(8) = 126.4; *P* < 0.001). Most farmers from Addis Ababa city (76%) indicated that butchery was their primary source of meat, compared to only 6.9% among farmers from Gondar in the Amhara region. Also mean age and the primary source of meat were found to be statistically different (F = 4.15; *P* = 0.016) between those households using butchery (45 years) as a primary source and those using home slaughter as a primary source (49 years). The results indicated that none of the other socioeconomic factors were associated to the primary meat source of a household.

#### Knowledge of zoonoses

The interviewed farmers were also asked if they think that eating raw meat can cause diseases. The result shows that the vast majority of farmers (92.9%) believed that consumption of raw meat can cause diseases and about 40% had actually experienced disease symptoms which they attributed to eating raw meat (Table 3D). Many respondents reported that they had experienced diseases and symptoms after eating raw meat, such as abdominal discomfort, tape worm, amoeba, gout and even TB.

Farmers were also asked if they knew that TB can be transferred from animals to humans through consumption of raw meat. Out of 477 respondents, 62.3% (297) indicated that they thought eating raw meat could do so, while 23.1% (*n* = 110) indicated that they did not know whether this was the case. Only 6.3% (*n* = 30) stated that TB cannot be transferred from animal to human by eating raw meat.

We also investigated whether a relationship exists between having attended training on zoonotic diseases and bTB transmission pathways, and farmers’ meat consumption behaviour. The results of this test indicated that there is a statistically significant relationship (likelihood-ratio chi2 (2) = 7.72; *P* = 0.021). Among the 348 farmers who had not undertaken training on zoonoses provided by local government extension services, 33.3% indicated that they consumed raw meat frequently. In contrast, only 24.3% of the 123 farmers who undertook training on zoonoses indicated that they consumed raw meet frequently. Our data also show that there was a statistically significant relationship between raw meat consumption habit and past occurrence of TB in the family (likelihood-ratio chi2 (2) = 5.68; *P* = 0.017). Out of the 48 farm households who reported that there has been a confirmed human TB case in the last 3 years in their farm, 20.8% indicated that they have the habit of raw meat consumption while the 79.2% for those farm households reported no TB case in the past 3 years.

### Determinants of raw milk and meat consumption

#### Determinants of raw milk consumption frequency

The results of this analysis indicate that the independent variables in our model are good predictors of the frequency of raw milk consumption (LR chi square of 109.2, significant at 1% confidence level; *P* < 0.001 and Pseudo R-square of 0.184). Among the variables entered into the model, we found study site, gender of the household head, previous animal bTB testing in farm, knowledge of zoonotic risk of milk consumption, household size, and per-capita milk consumption levels were important determinants of frequency of raw milk consumption among the studied dairy farm households (Table 4).

**Table 4:** Generalised Ordered Logit Estimates of raw milk (Eq. 1 and Eq2) and meat (Eq3 and Eq4) consumption frequency among dairy farmers in Ethiopia

	Raw milk consumption frequency		Raw meat consumption frequency	
VARIABLES	Eq1 (A vs B + C)	Eq2 (A + B vs C)	Eq3 (D vs E + F)	Eq4 (D + E vs F)
Oromia towns around Addis Ababa	0.508*	0.923**	0.584**	0.395
	(0.296)	(0.391)	(0.297)	(0.304)
Gondar	−0.978**	− 0.372	− 0.257	1.211***
	(0.491)	(0.630)	(0.395)	(0.410)
Mekele	−17.45	21.66	−2.146***	−2.324***
	(1318)	(5068)	(0.439)	(0.574)
Hawassa	0.794	0.555	0.644	−0.221
	(0.483)	(0.566)	(0.528)	(0.565)
Sex (1 = male; 0 = Female)	0.628*	−0.176	0.743***	0.448
	(0.33)	(0.436)	(0.281)	(0.328)
Meat preference (1 = beef; 0 otherwise)	–	–	0.507**	0.212
	–	–	(0.251)	(0.264)
Literacy (1 = Literate; 0 otherwise)	0.0833	0.929	0.339	0.315
	(0.555)	(0.733)	(0.469)	(0.558)
Can raw meat cause TB (1 = yes; 0 otherwise)	–	–	−2.287***	−1.120**
	–	–	(0.694)	(0.521)
Raw milk cons. Has zoonotic risk (1 = Yes)	− 0.412	− 1.745***	–	–
	(0.413)	(0.531)	–	–
Previous bTB Test (1 = Yes; 0 otherwise)	− 0.728**	−1.575***	− 0.401	− 0.373
	(0.307)	(0.481)	(0.260)	(0.284)
Know benefits of pasteurisation (1 = Yes)	−0.117	0.0879	–	–
	(0.277)	(0.340)	–	–
Had any zoonosis training (1 = yes; 0 otherwise)	0.0656	0.698	0.0626	−0.593**
	(0.305)	(0.442)	(0.269)	(0.290)
Herd size in number	–	–	−0.00170	−0.00929
	–	–	(0.00503)	(0.00678)
Number of milking cows	−0.00236	0.000149	–	–
	(0.00174)	(0.00280)	–	–
Per capita milk consumption per day	0.768*	1.086**	–	–
	(0.435)	(0.488)	–	–
Meat per capita consumption per month in kg	–	–	0.206	0.828***
	–	–	(0.138)	(0.145)
Age of the household head	−0.0152	−0.00135	0.0265	0.0908*
	(0.0101)	(0.0147)	(0.0505)	(0.0537)
Age squared	–	–	−0.000424	−0.000939*
	–	–	(0.000484)	(0.000523)
Household size	0.106*	0.190**	0.0203	0.159**
	(0.0602)	(0.0751)	(0.0591)	(0.0631)
Constant	−0.633	−2.401*	1.266	−4.167***
	(0.968)	(1.431)	(1.471)	(1.496)
Observations	440	440	417	417

Standard errors in parentheses; *** *p* < 0.01, ** *p* < 0.05, * *p* < 0.1

The results of the model suggest that, as compared to farm households in Addis Ababa, being in the Oromia towns surrounding Addis Ababa increased both the probability of raw milk consumption as well as its frequency. Being from Gondar decreased the probability of a respondent consuming raw milk.

The gender of the household head was also found to be an important determinant of raw milk consumption habits. Our results indicate that a household head being male increased the probability of raw milk consuming, but not the frequency of that consumption.

Awareness of bTB due to previous testing of cattle at farm decreased both the probability of raw milk consumption and its frequency. Its effect was more pronounced on decreasing the frequency of consumption, indicating that although there has been change in behaviour regarding raw milk consumption due to awareness of a farm’s bTB status, this change seems to have impacted more in decreasing the frequency at which raw milk was consumed, rather than halting its consumption altogether. In addition, knowledge of the possible zoonotic risk associated with raw milk consumption had its own effect on the raw milk consumption behaviour of farmers, in that it was found to significantly decrease the frequency of raw milk consumption but not the probability that raw milk would be consumed at all.

Household size and per capita milk consumption were found to be important determinants of raw milk consumption habits. With an increase in household size, the probability of raw milk consumption and the frequency of raw milk consumption were found to increase significantly. Similarly, with higher per capita milk consumption, both the probability of raw milk consumption and its frequency were found to increase significantly. The reasons behind this are not clear but could be due to the probable increased costs associated with boiling more milk or purchasing more pasteurised milk consumed by more people per capita.

#### Determinants of raw meat consumption frequency

The results of the analysis (Table 4) indicate that some of the independent variables in our model are good predictors of the frequency of raw meat consumption (LR chi square = 156.3, *p*-value = 0.000 (SD = 99%)). Study site, gender of household head, knowledge about zoonotic risks associated with raw meat consumption, training on zoonoses, age squared, household size, and per capita consumption of meat (of all types, either raw or cooked) were all found to be significant variables when predicting the frequency of an individual’s raw meat consumption, i.e. which of the three stated categories (D-F) they would fall into.

In terms of study site as a predictor, respondents based in the Oromia towns surrounding Addis Ababa were more likely to consume raw meat than those in Addis Ababa city, however, the frequency of such consumption was not significant. As compared to respondents from the capital, an average household based in Gondar did not consume more raw meat, but dairy households in Gondar tended to consume raw meat more frequently than those in Addis Ababa. However, the data also indicates that being based in Mekele reduced both the probability of consumption of raw meat as well as its frequency.

The gender of the household head was also found to affect the probability of raw meat consumption, but not the frequency of that consumption; a household head being male significantly increased the probability of members of that household consuming raw meat. Training on zoonotic disease transmission risks was found to have an effect on the frequency, rather than the probability of consumption, meaning those households which had access to zoonosis training tended to report a lower frequency of raw meat consumption as compared to those who did not have access to zoonosis training.

The result showed that age of dairy farmers had a positive effect on raw meat consumption frequency up to some limits but the effect of age on the frequency of raw meat consumption turned to be negative as farmers got older. However, age did not have a significant effect on the probability of raw meat consumption. Young farmers tended to have higher raw meat consumption frequency and as farmers got old they tended to decrease the frequency of raw meat consumption.

Interestingly, having ‘knowledge of the effects of raw meat consumption on the risk of zoonotic transmission of diseases’ had a statistically significant effect on both the probability of raw meat consumption and its frequency, and with a higher impact on the former. This means having knowledge about the risks involved in consumption of raw meat negatively affected both the decision to consume raw meat as well as its frequency, but it affected the former much more than the later.

Our data also suggests that the effect of meat type preference, i.e. a farmer who preferred beef meat, also had positive and significant effect on the probability of eating raw meat; however, no effect on the probability of the raw meat consumption frequency was seen. This might be due to the suitability of beef meat for raw meat-based meals such as *kitfo*, *kurt,* and *gored gored*, their local names in the Amharic language.

In the model, erd size was entered as a proxy variable to capture the effect of wealth on raw meat consumption habits. The result indicated that the habit of consuming raw meat was similar across the different wealth categories in Ethiopia and there was no statistically significant difference between the behaviour of the rich and the poor in this regard.

The data showed that high consumption of meat in general (expressed as ‘Per capita meat consumption’) did not affect the probability of raw meat consumption but it did increase the probability of doing so more frequently. Increased family size was also found to be linked with increased frequency of raw meat consumption.

## Discussion

### Milk consumption

Compared to the national average, the higher per capita milk consumption observed in this study is not surprising as we surveyed dairy farmers who should have better access to milk and who are likely to consume more milk than the general public. About 20% of the study population had the habit of drinking raw milk at least once a month while the vast majority drank the milk boiled. As many as a 25% drank boiled milk on a daily basis while nearly 90% drank it on a weekly basis. Less than 10% drank pasteurised milk with any frequency. Although nearly four out of five sampled farmers said that they never drank raw milk, we found that over 80% consumed fermented milk, the *ergo* yoghurt. This result is similar to other studies that also found high rates of yoghurt consumption in Ethiopia, especially among adults [49, 50]. Whether *ergo* can still contain live pathogens such as *M. bovis* after being fermented has yet to been proven, but a study from South Africa have shown that *M. bovis* can survive in both fresh and souring milk for periods of time that represent a risk of exposure to people consuming these products. However, the conditions for survival were dependent on both storage temperature and dose of pathogen in the milk products [51]. In Ethiopia, raw milk consumption is a common practice in various parts of the country. According to Negash et al. [52], 50% of the milk produced by smallholder farmers in the Ethiopian Rift Valley areas was consumed at home, in its fresh form, without being boiled or pasteurised. According to Ayele et al. [53], 35% of dairy farmers included in a survey around Sebeta in central Ethiopia, which is also one of the study areas in the current study, indicated that they drank raw milk and only 13% of these farmers were aware of food borne diseases, which can be transmitted through drinking raw milk. Another study showed that as many as 67% of the interviewed farmers in North Western Ethiopia drank raw milk [54]. Tolosa et al. [50] also indicated that in the Jimma area of Western Ethiopia, 57% of the adults drank fermented milk sporadically and 14% of the interviewees did not boil the milk for their children. Therefore, these findings suggest that some considerable proportion of the society in Ethiopia drink raw milk in its fresh or fermented form, and if not on a regular basis, at least occasionally. In comparison to these figures, our result showed a relatively low level of raw milk consumption frequency, possibly because our study sites were urban and peri-urban areas where people have better access to information regarding the zoonotic risks associated with drinking raw milk.

Among all socioeconomic variables studied, the statistically significant systematic relationship seen between study site and raw milk consumption habit implies that, rather than demographic variables such as sex and literacy, differences in raw milk consumption by study site might be related to differences in facilities available for milk processing in different locations across Ethiopia. Absence of any relationship between training on zoonoses given and raw milk consumption could suggest that the training given were not adequate for precipitating behavioural change among the people, and/or because of their positive perceptions of nutritional qualities, good taste or health benefits of consuming raw milk as indicated by Oliver et al. [17]. The findings of this study on farmers’ perception of the healthiness of drinking raw milk is in line with a previous study that suggested that, even in cases where a considerable proportion of the society have knowledge of zoonotic diseases, the practice of consumption of boiled or pasteurised milk was found to be low [16]. This could be related to the fact that although people are aware of the risk of infection, they may not always notice an infection after drinking raw milk. In the case of bTB in particular, transmission of the causative agent *M. bovis* may occur from unprocessed milk, but an infected person may only develop a latent infection and not display apparent clinical symptoms until later in life. In those cases, people may not associate TB with their raw milk consumption and they may also ignore their awareness of the associated risk and continue drinking raw milk, especially if they believe that it also has positive qualities, such as a superior taste.

The low level of pasteurised milk consumption in our study is likely associated with the under-developed milk pasteurisation practice in Ethiopia which has resulted in that the vast majority (95%) of all milk produced in the country is sold through informal marketing systems without passing through pasteurisation plants [55]. In such informal market systems, farmers often sell their milk directly to consumers and there is no mechanism for regulating bacteriological quality standards of the milk. Peoples’ knowledge of the importance of pasteurisation is also limited. According to Girma [56], in a survey conducted in North Shewa area in Ethiopia, only 3.5% of the respondents knew about pasteurisation as a means of preventing milk borne zoonosis. The high level of boiled milk consumption practice in this study is in line with the findings by Lemma et al. [57] and Duguma and Janssens [49]. However, the difference in frequency of boiled milk consumption by study site could be due to differing levels of awareness about the prevalence and health impacts of bTB and other zoonotic diseases, but would warrant further study.

According to the results of our generalised ordered logit model there are differences in milk consumption behaviour across study areas which might be attributed to differences in access to relevant information and pasteurised milk, as well as differences in perceived risk of contracting diseases due to raw milk consumption. Further studies could usefully investigate the detailed knowledge of farmers in different areas, as well as documenting what kinds of training they have received. The regional differences on milk consumption should also be noted by policy makers seeking to design cost-effective strategies for preventing and controlling zoonotic diseases through public behaviour change and/or investment in milk pasteurisation technology.

Our analysis suggests that awareness and knowledge about bTB and/or other zonootic risks of consuming raw milk had the effect of decreasing raw milk consumption but without stopping the consumption completely. This could be due to perceptions about the nutritional qualities, good taste or health benefits of raw milk, but it could also be due to the fact that raw milk was easily, quickly, and conveniently available to the population that we surveyed. Boiling, fermenting, or pasteurising milk takes time and energy and may sometimes be abandoned in favour of the most easily available option of drinking the milk raw, despite the associated risks. This implies that repeated and multichannel education and information dissemination need to be given in order to bring about permanent behavioural change among farmers in terms of raw milk consumption.

### Meat consumption

Our overall finding that the per capita meat consumption across the sampled farmers was considerably higher than the national average was not surprising, given that our study sites were all located in urban or peri-urban areas where meat consumption rates tend to be higher [58]. Within this population, with an overall higher meat consumption, households with female heads were found to consume less meat per capita than those with male heads. This may be attributed to differences in income between those with household heads of different genders and/or different nutritional priorities, but it was not possible to investigate this further using our current dataset. In this regard, it should be added that whilst it might be expected that those households with literate heads might have higher per capita meat consumption on account of potentially having higher incomes, this was not the case in our sample.

In terms of meat source preference, we found that those farmers living in Hawassa, Addis Ababa city, and the Oromia towns surrounding Addis Ababa were considerably more likely to prefer beef as their first-choice meat, while those in Gondar and Mekelle were more likely to prefer mutton. This difference can be linked to the relatively slow development of beef abattoirs and associated industry in the Amhara and Tigray regions, as compared to in Addis Ababa and nearby areas in central Ethiopia, as well as in Hawassa in the southern part.

Overall, the sampled farmers favoured butcheries, who tend to source their meat from abattoirs, as their first choice for sourcing meat. However, location or study site affected their expressed preferences surrounding meat sources. A significant majority of farmers in Addis Ababa, over 75%, stated that they preferred to source meat from butchers, but this proportion was extremely low, at only 6.9%, in Gondar. This finding is also likely to be related to butcheries in Addis Ababa being both greater in number and in their capacity. It is also possible that those farmers who live in the growing metropolis of Addis Ababa, where land is at a premium, may have less space in which to slaughter animals themselves.

The results of the analysis using the generalised ordered logit model showed relationships between a number of variables and both the probability of any raw meat consumption and also the frequency of raw meat consumption amongst those who reported that they were in the habit of eating raw meat. Identifying such relationships may prove useful to policy-makers and veterinary and medical professionals seeking to understand and influence raw meat consumption behaviours in Ethiopia, particularly in the context of preventing and controlling the transmission of zoonotic diseases, of which raw meat consumption is a recognised risk factor (e.g. [59–61]).

In terms of study site, our findings suggest that farmers living in the Oromia towns surrounding Addis Ababa are more likely to consume raw meat than those in the capital itself. We also found that those farmers who do eat raw meat are more likely to do so at high frequency if they live in Gondar. For policy-makers and public health officials seeking to encourage reduced raw meat consumption, these findings suggest that their resources might be best used in the Oromia towns and in Gondar, as opposed to in Addis Ababa, or indeed in Mekelle, where raw meat consumption is lower. However, as it is likely that raw meat consumption rates in Mekelle are lower because of the relatively underdeveloped beef abattoir industry in Mekelle, it would be sensible to monitor raw meat consumption in that town as the industry develops, as meat type preferences are likely to shift as a result.

Across the different research study sites, the results of the generalised ordered logistic model analysis show that older people are less likely to consume raw meat at high frequency. This may be due to people accumulating knowledge of the health risks surrounding raw meat consumption over the life course and/or a general shift in diet as people age. In focus group discussions carried out as part of the ETHICOBOTS project, dairy farmers in the Oromia towns around Addis Ababa reported that some older people no longer consume any meat because it is believed to speed up the aging process [30]. With the assumption that the study areas represent urban and peri-urban centres in Ethiopia, the result indicates that being vegetarian in Ethiopia is not a common habit (0.6%). Most people eat meat, yet they do not eat much. The reasons behind the low level of vegetarianism might be that the generally low level of meat consumption did not yet cause higher prevalence of obesity an associated health risks in Ethiopia which in turn made people not to be serious about watching diets and adopt the habit of vegetarianism.

Although having received training on zoonoses did not seem to affect the probability that someone ever ate raw meat, it was found that having knowledge of the specific risks of zoonotic transmission from eating raw meat did have a statistically significant effect on both the probability of raw meat consumption and its frequency. In fact, our data show that it had a higher impact on the probability of consumption of raw meat than the probability of high frequency of raw meat consumption. This indicates that while training on zoonotic disease control in general seemed to have an impact of the amount of raw meat eaten, special care should be taken to include information about risks associated with raw meat consumption in these trainings. Further training could play an important role in shaping consumption behaviours and consequently represents a solid investment for public health policy makers concerned about transmission of disease through raw meat, due to poor inspection or handling of infected carcasses [62].

Among| those who reported a confirmed TB case in humans in their family in the last 3 years prior to our survey, 82.8% indicated a high raw meat consumption frequency, suggesting that the consumption of raw meat may increase the risk of TB transmission in Ethiopia. However, it should also be noted that 61.2% of those respondents with no reported TB cases in the family also consumed raw meat at high frequency. Those delivering training on the risks associated with consuming raw meat should therefore also pay attention to the cultural value of such practices and communicate to trainees that a risk of disease transmission by no means represents a certainty of that transmission. If this subtlety is not effectively conveyed, it is possible for distrust to emerge between trainers and trainees who, going on their own experience of contact with supposed risk factors without any obvious harm, may come to the conclusion that the risk is not genuine. It should also be made clear that the consumption of raw meat is not only associated with active, confirmed TB cases, but also with latent TB, which may not show symptoms for many years [61].

## Conclusions

### Implications for prevention and control of Zoonoses

The present study interviewed nearly 500 urban and peri-urban dairy farmers in major towns of Ethiopia about their milk and meat consumption patterns to understand the potential risks of zoonotic disease transmission.

On their milk consumption behaviours, although consumption of heat-treated milk was the most common, we concluded that around 20% of the study population still drank raw milk at least on a monthly basis. Given the high prevalence of bTB in the cattle population in the explored study areas, the habit of raw milk consumption may expose farmers to zoonotic TB transmission. Despite the need for additional clinical enquiry, empirical evidence from this study suggest that there was a statistically significant link between self-reported TB infection amongst humans on the farm and the bTB status of its cattle. Therefore, bTB control strategies should aim to raise awareness among the dairy farming as well as in the general population of the possible zoonotic risks involved in raw milk consumption and the importance of pasteurisation and milk boiling in mitigating these risks. Moreover, variables such as location, gender, household size and per capita milk consumption need to be considered in any effort to induce voluntary behavioural change surrounding raw milk consumption habits to tackle the risks of transmission of zoonotic diseases.

With regards to meat consumption behaviours, given that consumption of raw meat presents a risk of contracting diseases such as bTB, salmonella, taeniasis and others through zoonotic transmission, and that frequent consumption increases this risk, the urban and peri-urban dairy farming population are exposed to a considerable level of zoonotic risk. However, the level of exposure to such risks may vary from town to town and is based on disease prevalence, local cultural orientations, and relative availability of infrastructure in place, such as abattoir services including their meat inspection praxises. It should also be stressed that, as most animals in Ethiopia are likely to not be slaughtered based on controlled hygienic practices, but rather at home or at communal slaughtering, the risk of contaminating the meat used for consumption during slaughter, and thereby the risk of zoonotic transmission, increases. Increased capacity for slaughter at controlled abattoirs as well as improvement of routine meat inspections at such abattoirs are therefore recommended.

Besides designing control strategies for reducing disease prevalence in animals at farm level in general, creating risk awareness about zoonotic disease transmission to consumers through training and media campaigns, increase availability of pasteurised or heat-treated milk, improving meat hygiene through better abattoir services, and inducing behavioural change around meat sourcing and raw meat consumption, are all crucial to the successful prevention and control of the spread of zoonotic diseases, including bTB.

## Supplementary Information

**Additional file 1.**

**Additional file 2.**

## Data Availability

All raw data collected and used for this study are available as supplementary material.
