# Supplementary material for: Milk and meat consumption patterns and the potential risk of zoonotic disease transmission among urban and peri-urban dairy farmers in Ethiopia
Source: BMC Public Health. 2022 Feb 3;22:222. doi: 10.1186/s12889-022-12665-4 (PMC8815239; doi:10.1186/s12889-022-12665-4)
Supplement: Supplementary file 1 — Additional file 1. [file 12889_2022_12665_MOESM1_ESM.docx]

**Supplementary Table S1**. Distribution of Per-capita milk consumption per day (in litres) by different socioeconomic variables

| **Variable** | **Values** | **N** | **Mean** | **Std. dev.** | **t/F value** | **P Value** |
| --- | --- | --- | --- | --- | --- | --- |
| Respondent's sex | Female | 113 | 0.22 | 0.16 | t = -1.15 | 0.25 |
|  | Male | 366 | 0.26 | 0.28 |  |  |
| Study site | Addis Ababa | 164 | 0.27 | 0.33 | F= 1.61 | 0.17 |
|  | Oromia towns | 136 | 0.25 | 0.21 |  |  |
|  | Gondar | 58 | 0.20 | 0.23 |  |  |
|  | Mekele | 59 | 0.20 | 0.14 |  |  |
|  | Hawassa | 25 | 0.27 | 0.17 |  |  |
| Literacy | Illiterate | 34 | 0.19 | 0.21 | t = -1.23 | 0.22 |
|  | Literate | 446 | 0.25 | 0.26 |  |  |
| Religion | Muslim | 20 | 0.18 | 0.16 | t= -1.04 | 0.30 |
|  | Christian | 458 | 0.25 | 0.26 |  |  |
